# Completion of Recommended Tests and Referrals in Telehealth vs In-Person Visits

**DOI:** 10.1001/jamanetworkopen.2023.43417

**Published:** 2023-11-15

**Authors:** Anthony Zhong, Maelys J. Amat, Timothy S. Anderson, Umber Shafiq, Scot B. Sternberg, Talya Salant, Leonor Fernandez, Gordon D. Schiff, Mark D. Aronson, James C. Benneyan, Sara J. Singer, Russell S. Phillips

**Affiliations:** 1 Center for Primary Care, Harvard Medical School, Boston, Massachusetts; 2 Division of General Medicine and Primary Care, Beth Israel Deaconess Medical Center, Boston, Massachusetts; 3 Division of General Medicine, Brigham and Women’s Hospital, Boston, Massachusetts; 4 Healthcare Systems Engineering Institute, Northeastern University, Boston, Massachusetts; 5 Stanford University School of Medicine, Stanford, California

## Abstract

**Importance:**

Use of telehealth has increased substantially in recent years. However, little is known about whether the likelihood of completing recommended tests and specialty referrals—termed *diagnostic loop closure*—is associated with visit modality.

**Objectives:**

To examine the prevalence of diagnostic loop closure for tests and referrals ordered at telehealth visits vs in-person visits and identify associated factors.

**Design, Setting, and Participants:**

In a retrospective cohort study, all patient visits from March 1, 2020, to December 31, 2021, at 1 large urban hospital-based primary care practice and 1 affiliated community health center in Boston, Massachusetts, were evaluated.

**Main Measures:**

Prevalence of diagnostic loop closure for (1) colonoscopy referrals (screening and diagnostic), (2) dermatology referrals for suspicious skin lesions, and (3) cardiac stress tests.

**Results:**

The study included test and referral orders for 4133 patients (mean [SD] age, 59.3 [11.7] years; 2163 [52.3%] women; 203 [4.9%] Asian, 1146 [27.7%] Black, 2362 [57.1%] White, and 422 [10.2%] unknown or other race). A total of 1151 of the 4133 orders (27.8%) were placed during a telehealth visit. Of the telehealth orders, 42.6% were completed within the designated time frame vs 58.4% of those ordered during in-person visits and 57.4% of those ordered without a visit. In an adjusted analysis, patients with telehealth visits were less likely to close the loop for all test types compared with those with in-person visits (odds ratio, 0.55; 95% CI, 0.47-0.64).

**Conclusions:**

The findings of this study suggest that rates of loop closure were low for all test types across all visit modalities but worse for telehealth. Failure to close diagnostic loops presents a patient safety challenge in primary care that may be of particular concern during telehealth encounters.

## Key Points

**Question:**

Is there a difference in the rate of completion for diagnostic tests and referrals ordered during a telehealth visit compared with those ordered during an in-person visit?

**Findings:**

In this cohort study of 4133 diagnostic tests and referrals (colonoscopies, cardiac stress tests, and dermatology referrals) ordered between March 1, 2020, and December 31, 2021, at 2 affiliated clinical primary care sites, 58% of those ordered during in-person visits were completed within the designated time frame compared with 43% of those ordered during telehealth visits. The rate of completion was between 40% and 65% for all test types, regardless of visit modality.

**Meaning:**

The findings of this study suggest that rates of completion for diagnostic tests and referrals were low for all visit types but worse when ordered during telehealth visits.

## Teaser

This cohort study examines the rates of completion of referrals for tests among patients with telehealth, in-person, and no visits.

## Introduction

The use of telehealth has increased substantially in recent years, by more than 60-fold in 2020.^1^ Although telehealth made up less than 1% of medical visits before the COVID-19 pandemic, it became ubiquitous with the onset of the pandemic, before tapering to still-unforeseen levels, with 37% of adults reporting at least 1 telephone or video visit in 2022.^2^ Past studies have examined patient experiences with telehealth, finding a high degree of satisfaction due to the ease of use, improved communication, and reduced wait and travel times.^3,4,5,6^ Studies have also found high levels of diagnostic concordance (defined as degree of agreement) overall between telehealth and in-person visits.^7,8^ However, little is known about how telehealth affects a patient’s likelihood of completing tests and specialty referrals—termed *diagnostic loop closure*.^9^

Failure to close referral loops is one of the leading causes of diagnostic errors in primary care. This is of particular importance with telehealth, given the finding of reduced diagnostic concordance^10^ in primary care due to the breadth of potential diagnoses.^7,9^ The eventual outcomes of these oversights can be catastrophic; an estimated 12 million diagnostic errors occur annually in the US, contributing to 64 000 preventable deaths and costing the health care system $100 billion per year.^11,12^

In this study, we examined the prevalence of the failure to close diagnostic loops for 3 representative types of tests and referrals ordered during primary care telehealth visits compared with those ordered during in-person visits and those ordered without a visit. We hypothesized that orders placed during telehealth visits were less likely to be completed due to a variety of patient, clinician, and system-level factors, which we sought to explore further.

## Methods

### Design, Setting, and Participants

We conducted a retrospective cohort study at 2 primary care sites in Boston, Massachusetts: 1 large urban hospital-based primary care practice and 1 affiliated community health center. We examined all visits from March 1, 2020, to December 31, 2021. The hospital-based site cares for approximately 42 000 patients while the affiliated community health center cares for approximately 6000 patients. We accessed clinical data for the 2 sites through a data warehouse, which maintains data for both and allows search by billing diagnoses, tests, referrals, and patient demographic characteristics. At both sites, patients have the option of both in-person visit and telehealth visits (telephone or video). Our research protocol was approved by the institutional review boards at Beth Israel Deaconess Medical Center and Harvard Medical School. The institutional review boards at both institutions deemed that informed consent from study participants was not required because only deidentified data were involved. This study followed the Strengthening the Reporting of Observational Studies in Epidemiology (STROBE) reporting guideline.^13^

We reviewed referrals and tests resulting from a concerning finding or need for screening, prompting the clinician to order an additional diagnostic workup. The order may have been placed during an in-person visit, a telehealth visit, or on a date when no visit occurred. We extracted data for 3 types of high-risk tests and referrals ordered between March 1, 2020, and December 31, 2021: colonoscopy referrals, dermatology referrals for suspicious skin lesions, and cardiac stress tests (including both exercise and chemical stress tests, with and without imaging). These tests were chosen due to their different characteristics, ease of test completion, high-risk nature with major clinical implications, and institutional prioritization. We then examined all orders for completion between March 1, 2020, to March 30, 2022.

Within all visit modalities, the order for testing or referrals is placed electronically through the electronic medical record. There is little to no proactive outreach for the scheduling of patients for routine orders. During an in-person visit, the patient is given a form with a telephone number to call to schedule the test or referral. For patients with limited English proficiency or complex needs, a member of the support staff team may help the patient schedule this test at checkout. During a telehealth visit, a patient is given the telephone number to call to schedule the test or referral by the clinician during the visit. In all scenarios, the patient receives no communication after the visit reminding them to schedule the test or referral.

### Main Measures

Our main independent variable was the modality of the patient visit during which the diagnostic test or referral was ordered (in-person, telehealth [telephone and video], or ordered without a visit). Our primary outcome was failure to close the loop on the test or referral. The loop was considered closed if the orders were completed within 365 days for colonoscopy, 90 days for dermatology, and 45 days for cardiac stress test referrals. Time intervals for loop closure were determined through review of the literature, in conversations with physicians administering the tests based on the urgency of making a diagnosis, and with the goal of ensuring adequate time for patients to schedule despite current systematic delays.

We included several patient factors obtained from the electronic medical record as independent covariates. These included demographic variables such as age, sex assigned at birth (female or male), self-reported race (Asian, Black, White, or unknown or other), self-reported ethnicity (Hispanic, non-Hispanic, or unknown), educational level (high school or less, college, or unknown), primary language (English, other, or unknown), and insurance type (Medicaid, Medicare, commercial, or unknown or other), which we regarded as a surrogate for income level. Race and ethnicity were included due to the importance of identifying disparities in care as a first step toward addressing them. Race was divided into the following categories: American Indian or Alaska Native, Asian, Black or African American, White, declined to answer, other race or mixed race, prefer not to say, unable to obtain, and unknown or not specified. For ease of reporting, it was narrowed down (Table 1). The unknown or other category includes American Indian or Alaska Native, declined to answer, other race or mixed race, prefer not to say, unable to obtain, and unknown or not specified.

**Table 1.** Patient Characteristics by Visit Modality

Patient characteristic	No. (%)				*P* value^a^
	Overall (N = 4133)	In-person (n = 2167 [52.4%])	Telehealth (n = 1151 [27.8%])	No visit (n = 815 [19.7%])	
Age, y					
Mean (SD)	59.3 (11.7)	59.5 (11.6)	58.6 (10.8)	59.5 (13.2)	.09
Median (IQR)	60 (51-67)	60 (51-68)	59 (51-66)	61 (51-68)	
Sex					
Female	2163 (52.3)	1141 (52.7)	640 (55.6)	382 (46.9)	<.001
Male	1970 (47.7)	1026 (47.3)	511 (44.4)	433 (53.1)	
Race^b^					
Asian	203 (4.9)	121 (5.6)	46 (4.0)	36 (4.4)	<.001
Black	1146 (27.7)	672 (31.0)	299 (26.0)	175 (21.5)	
White	2362 (57.1)	1147 (52.9)	701 (60.9)	514 (63.1)	
Unknown or other	422 (10.2)	227 (10.5)	105 (9.1)	90 (11.0)	
Ethnicity^b^					
Hispanic	353 (8.5)	195 (9.0)	80 (7.0)	78 (9.6)	.10
Not Hispanic	3533 (85.5)	1843 (85.0)	1009 (87.7)	681 (83.6)	
Unknown	247 (6.0)	129 (6.0)	62 (5.4)	56 (6.9)	
Educational level					
High school or less	1622 (39.3)	916 (42.3)	407 (35.4)	299 (36.7)	<.001
College	1993 (48.2)	979 (45.2)	597 (51.9)	417 (51.2)	
Unknown	518 (12.5)	272 (12.6)	147 (12.8)	99 (12.1)	
Primary language					
English	3626 (87.7)	1857 (85.7)	1048 (91.1)	721 (88.5)	<.001
Other	483 (11.7)	297 (13.7)	96 (8.3)	90 (11.0)	
Unknown	24 (0.6)	13 (0.6)	7 (0.6)	4 (0.5)	
Insurance					
Medicaid	799 (19.3)	464 (21.4)	204 (17.7)	131 (16.1)	.001
Medicare	1367 (33.1)	706 (32.6)	359 (31.2)	302 (37.1)	
Commercial	1882 (45.5)	954 (44.0)	566 (49.2)	362 (44.4)	
Unknown or other	85 (2.1)	43 (2.0)	22 (1.9)	20 (2.5)	
Medical complexity (Charlson comorbidity index score)					
Low (0)	2185 (52.9)	1143 (52.7)	615 (53.4)	427 (52.4)	.36
Medium (1-2)	1241 (30.0)	657 (30.3)	353 (30.7)	231 (28.3)	
High (≥3)	707 (17.1)	367 (16.9)	183 (15.9)	157 (19.3)	
Mental illness					
Presence of depression	776 (18.8)	410 (18.9)	213 (18.5)	153 (18.8)	.96
Primary care site					
Hospital-based primary care clinic	3730 (90.3)	1918 (88.5)	1064 (92.4)	748 (91.8)	<.001
Affiliated community health center	403 (9.8)	249 (11.5)	87 (7.6)	67 (8.2)	
Patient engagement					
Use of patient portal	1392 (33.7)	672 (31.0)	397 (34.5)	323 (39.6)	<.001
Ordering clinician type					
Attending physician	3312 (80.1)	1664 (76.8)	970 (84.3)	678 (83.2)	<.001
Nurse practitioner	236 (5.7)	157 (7.2)	26 (2.3)	53 (6.5)	
Resident	585 (14.2)	346 (16.0)	155 (13.5)	84 (10.3)	

^a^ For tests of statistical significance, analysis of variance was used for continuous variables and χ^2^ analysis was used for all other variables.

^b^ Full list of self-reported races, ethnicities, and national heritages. Race: American Indian or Alaska Native, Asian, Black or African American, White, declined to answer, other or mixed race, prefer not to say, unable to obtain, or unknown or not specified. Ethnicity or national heritage: African, African American, American, Asian, Asian Indian, Brazilian, British, Cape Verdean, Caribbean, Central American (not otherwise specified), Chicano, Chinese, Columbian, Dominican, Eastern European, European, Filipino, French, German, Guatemalan, Haitian, Honduran, Irish, Italian, Japanese, Korean, Mexican, Mexican American, Middle Eastern, Portuguese, Puerto Rican, Russian, Salvadoran, Scottish, South American (not otherwise specified), Ugandan, Vietnamese, West Indian, declined to answer, other, prefer not to say, unable to obtain, or unknown or not specified. Hispanic indicator: Hispanic, not Hispanic, or unknown or not specified.

We also included patients’ chronic illness burden and psychiatric illness burden as covariates. Chronic illness burden was calculated using the Charlson comorbidity index (0, 1-2, or ≥3).^14^ This was determined using the billed diagnosis codes for all medical visits within 2 years of the index referral date. Psychiatric illness burden was based on a billing code for depression (*International Statistical Classification of Diseases and Related Health Problems, Tenth Revision* code F32) at a visit before or on the index referral date, using the date range (yes or no). Other markers of psychiatric illness, such as anxiety, were not examined.

Other covariates described the clinical site (hospital-based primary care practice and affiliated community health center), type of clinician ordering the test or referral (attending physician, resident physician, or nurse practitioner), and whether the patient had ever used the patient portal to review a visit note, which we used as a surrogate for patient engagement. We additionally included a marker for pause of elective procedures at our institution to represent periods of high COVID-19 infectivity, which occurred from March 13 to June 9, 2020; December 7, 2020, to March 1, 2021; and November 21, 2021, to January 27, 2022.

### Statistical Analysis

We compared patient characteristics between the 3 groups: patients with orders placed during in-person visits, telehealth visits, and no visit. We then performed a univariate analysis with loop closure as the outcome. For the primary comparison of loop closures associated with telehealth visits with in-person visits, we adjusted for all the patient factors listed above using multivariable logistic regression. Additionally, we performed a stepwise regression analysis to identify the extent to which groups of covariates contributed to the findings (ie, we sequentially adjusted for grouped covariates to determine which group best explained the univariate result). The stepwise regression analysis first adjusted for the test type, then patient demographic characteristics and comorbidities, then clinical site and clinician type, and finally patient engagement. For tests of statistical significance, analysis of variance was used for continuous variables and χ^2^ analysis was used for all other variables. We conducted all statistical analyses using Stata, version 16.1 (StataCorp LLC), with statistical significance set at a 2-sided α value of .05.

## Results

### All Orders

Among 4133 patients with test and referral orders, the mean (SD) age was 59.3 (11.7) years; 2163 (52.3%) were women and 1970 (47.7%) were men; 203 (4.9%) were Asian, 1146 (27.7%) were Black, 2362 (57.1%) were White, and 422 (10.2%) had an unknown or other race (Table 1). The 4133 orders included colonoscopy referrals (n = 3254), dermatology referrals for suspicious skin lesions (n = 455), and cardiac stress tests (n = 424). Of these, 2167 (52.4%) were ordered during an in-person visit, 1151 (27.8%) were ordered during a telehealth visit, and 815 (19.7%) were ordered without a visit. Compared with patients with in-person visits, patients with telehealth visits were more likely to be White, have higher educational attainment, have English as their primary language, have commercial insurance, and to have been seen by an attending physician (Table 1).

**Figure 1.**
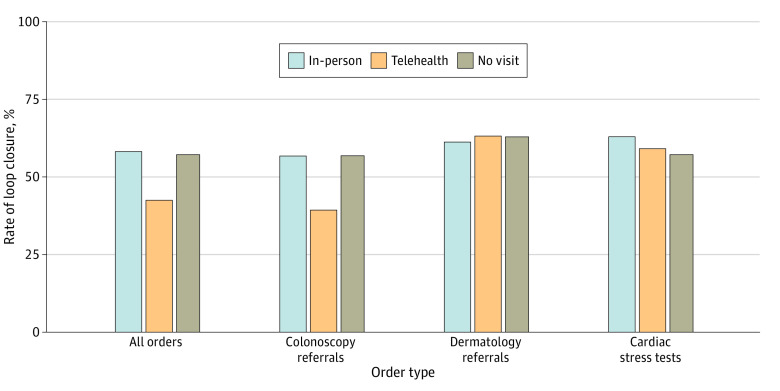
Rate of Loop Closure by Order Type and Visit Modality

**Figure 2.**
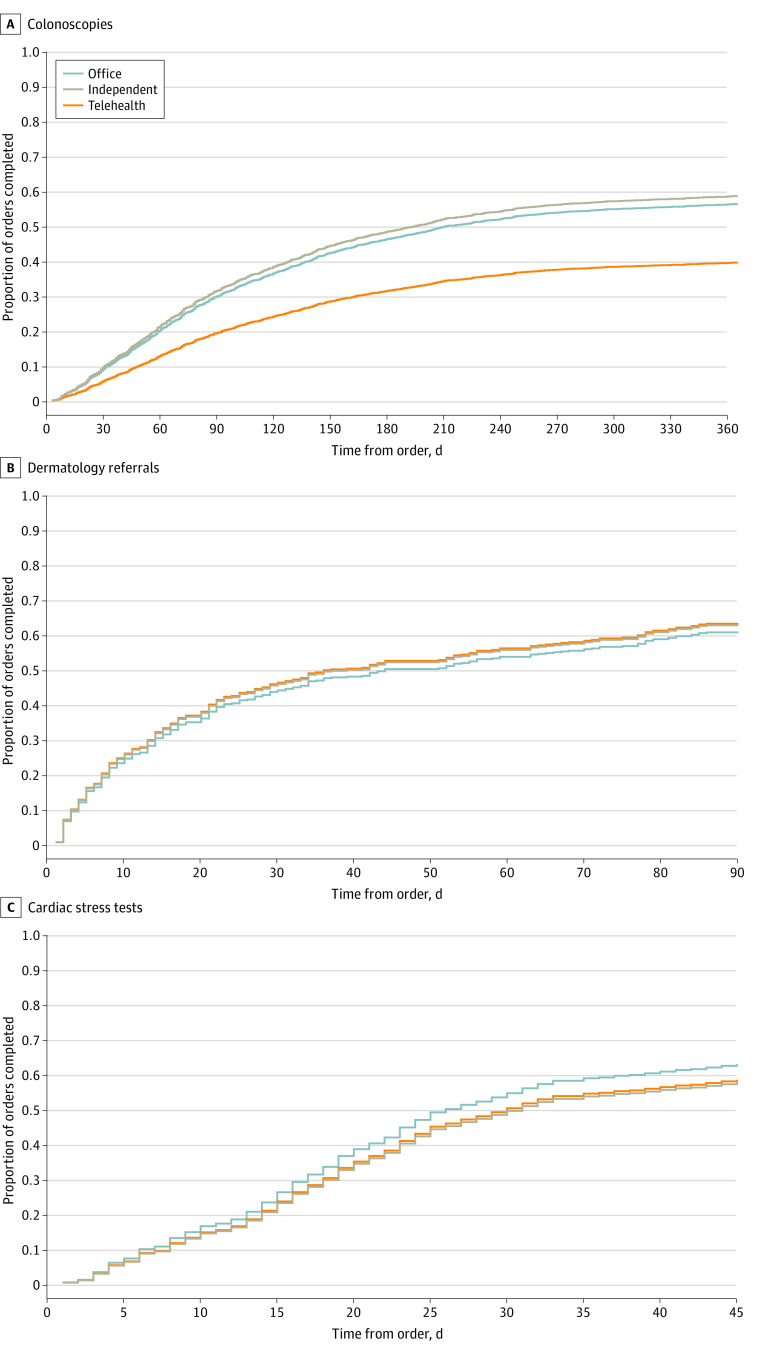
Time to Loop Closure by Test Type and Visit Modality

Of the 1151 orders placed during a telehealth visit, 490 (42.6%) were completed within the designated time frame vs 58.4% (1265 of 2167) placed during an in-person visit and 57.4% (468 of 815) placed without a visit (Figure 1). In the univariate analysis, patients with telehealth visits were less likely to close the loop on high-risk tests and referrals overall compared with in-person visits (odds ratio [OR], 0.53; 95% CI, 0.46-0.61) (Table 2). Patients with orders placed without an in-person or telehealth visit were equally likely as patients with an in-person visit to close the loop on ordered tests and referrals. A stepwise multivariable regression that adjusted for groups of covariates sequentially did not result in significant changes in rates of loop closure when comparing telehealth visits with in-person visits (Table 3). In the adjusted analysis, patients with telehealth visits were still less likely to close the loop on all tests and referral types overall (OR, 0.55; 95% CI, 0.47-0.64). When adjusting for periods of high COVID-19 infectivity, the overall odds of loop closure were 0.58 (95% CI, 0.51-0.67) across all referrals and test orders. The rate of loop closure plateaued before the allotted time frame expired for all test types (colonoscopies, 365 days; dermatology referrals, 90 days; cardiac stress tests, 45 days) and the time to loop closure did not appear to differ between orders originating from telehealth vs in-person visits (Figure 2).

**Table 2.** Odds of Loop Closure by Test Type for Telehealth Visits vs In-Person Visits

Analysis	Odds ratio (95% CI)			
	All orders	Colonoscopy	Dermatology	Stress test
Unadjusted	0.53 (0.46-0.61)	0.50 (0.43-0.59)	1.07 (0.61-1.87)	0.84 (0.52-1.37)
Adjusted^a^	0.55 (0.47-0.64)	0.49 (0.42-0.58)	1.07 (0.60-1.95)	0.84 (0.49-1.43)

^a^ Adjusted for all covariates, including age, gender, race and ethnicity, educational level, primary language, type of insurance, medical complexity, mental illness, primary care site, patient engagement, and ordering clinician type.

**Table 3.** Stepwise Multivariable Regression Analysis for All Covariate Group Models

Variable	Odds ratio (95% CI)
Unadjusted	0.53 (0.46-0.61)
Adjusted for test type	0.55 (0.48-0.64)
Adjusted for demographic characteristics and comorbidities	0.55 (0.48-0.64)
Adjusted for clinical site and clinician type	0.55 (0.47-0.64)
Adjusted for patient engagement	0.55 (0.47-0.64)
Final adjusted	0.55 (0.47-0.64)

### Colonoscopy Referrals

We identified a total of 3254 colonoscopy referrals, which made up 78.7% of all orders; 998 (30.7%) were ordered during a telehealth visit, 1570 (48.2%) were ordered during an in-person visit, and 686 (21.1%) were ordered without a visit. Of the colonoscopy referrals ordered at a telehealth visit, 39.8% (n = 397) were completed within 365 days, compared with 56.9% (n = 893) ordered at an in-person visit and 56.7% (n = 389) ordered without a visit. Before adjustment for the covariates, patients with telehealth visits were less likely to close the loop on colonoscopy referrals compared with in-person visits (OR, 0.50; 95% CI, 0.43-0.59). This remained consistent in the adjusted analysis (OR, 0.49; 95% CI, 0.42-0.58) (Table 2).

### Dermatology Referrals

We identified a total of 455 dermatology referrals for suspicious skin lesions, which made up 11.0% of all orders; 65 (14.3%) were ordered during a telehealth visit, 301 (66.2%) were ordered during an in-person visit, and 89 (19.6%) were ordered without a visit. Of the dermatology referrals ordered at a telehealth visit, 63.1% (n = 41) were completed within 90 days, compared with 61.5% (n = 185) ordered at an in-person visit and 62.9% (n = 56) ordered without a visit. There was no significant difference between telehealth and in-person visits for dermatology referral loop closure in the unadjusted (OR, 1.07; 95% CI, 0.61-1.87) or adjusted (OR, 1.07; 95% CI, 0.60-1.95) analyses (Table 2).

### Cardiac Stress Tests

We identified a total of 424 cardiac stress tests, which made up 10.3% of all orders; 88 (20.8%) were ordered during a telehealth visit, 296 (69.8%) were ordered during an in-person visit, and 40 (9.4%) were ordered without a visit. Of the stress tests ordered at a telehealth visit, 59.1% (n = 52) were completed within 45 days, compared with 63.2% (n = 187) ordered at an in-person visit and 57.5% (n = 23) ordered without a visit. There was no significant difference between telehealth and in-person visits for stress test loop closure in the unadjusted (OR, 0.84; 95% CI, 0.52-1.37) or adjusted (OR, 0.84; 95% CI, 0.49-1.43) analyses (Table 2).

## Discussion

Loop closure rates for selected diagnostic tests and specialty referrals were unacceptably low across all visit modalities. Furthermore, in a multivariable analysis, patients with telehealth visits were substantially less likely to close open loops compared with patients with in-person visits. In subgroup analyses based on test type, patients with telehealth visits were less likely to close the loop on colonoscopies, but statistically significant differences were not detected for urgent dermatology referrals or stress test orders. Sequentially adjusting for test type, patient demographic characteristics, comorbidities, clinical site, clinician type, and patient engagement did not markedly change the odds of loop closure in the stepwise regression analysis.

As reported, the rate of loop closure was low (between 40% and 65%) for all test types, regardless of visit modality, compared with an acceptable threshold of 80% in population health metrics and an ideal goal of 95% in models based on expert consensus.^15^ This is consistent with research that has found suboptimal completion rates for tests ordered in urgent care scenarios and suggests that common system factors may play a more important role in loop closure than visit modality.^16^ Thus, while the differences in loop closure between telehealth and in-person visits may be concerning, system-level changes are needed to improve test completion rates across all modalities. These might include automated tracking for outstanding tests within electronic medical records and interventions such as telephone outreach to patients, automated text and email reminders, and the use of referral managers.^17^ These considerations may be particularly important for patients who rely heavily on telehealth, such as those in remote rural areas and disadvantaged patients with limited health access and literacy.

When investigating notable differences in loop closure for orders placed during telehealth visits, our findings suggest that differences in loop closure may be inherent to telehealth as a modality. One potential mechanism to explain this may be the lack of systems in place to help patients complete test and referral orders. During in-person visits, members of the support staff team sometimes help patients schedule their tests at checkout; however, this support is absent during telehealth visits. After the visit, patients do not receive any communication reminding them to schedule the test or referral, which may further limit loop closure. Other potential explanations include the possibility that it may be more difficult to remember information provided during telehealth visits, that telehealth may present unique communication barriers, or that it may be more difficult to engage patients in shared decision-making during virtual visits, thus decreasing patient engagement with test and referral orders.^18,19,20,21^

These barriers may be more pronounced for colonoscopies compared with other types of orders given the increased difficulty in preparing for and completing the invasive procedure, thus explaining the disparities in loop closure compared with the other orders included in our analysis.^22^ Furthermore, given the notable difference in loop closure for colonoscopies, telehealth visits may serve as a marker for patients not participating in a high-complexity test. They may also highlight an important association between test indication and test completion, with screening indication yielding lower priority for both the patient and physician. This builds on past work that has identified lower rates of patient adherence to hemoglobin A_1c_ testing recommendations during telehealth encounters.^23^ With this association in mind, an order for a routine test placed during a telehealth visit might serve as a process marker to trigger additional assistance or alternative ways to complete the indicated test. Our observation that telehealth does not seem to worsen loop closure for tests prompted by patient symptoms or concerns (suspicious skin lesions or chest pain) suggests that patient activation may be a key factor in closing loops and may overcome some of the challenges inherent to telehealth visits.

The widespread failure to close diagnostic test and referral loops identified in this study may also provide a mechanism to explain past findings that primary care practices with higher telehealth use are associated with increased rates of acute care visits and that primary care telehealth visits are associated with increased emergency department encounters,^24,25^ possibly due, at least in part, to a failure to close the loop on ordered tests. These findings should be contextualized within the literature, which has identified significant advantages to telehealth vis-à-vis convenience, access, and diagnostic concordance.^5,6,7,8^ Nonetheless, our findings highlight a potential limitation to telehealth that, to our knowledge, had not been previously identified.

### Strengths and Limitations

Strengths of this study include the novelty of the inquiry, variety of tests evaluated, adjustment for confounding, and patient characteristics that are consistent with prior reports evaluating the composition of telehealth patient populations.^1,2^ This study has limitations. First, the study lacks data on the urgency of orders, scheduled tests and referrals that were canceled, and clinical outcomes resulting from the failure to close diagnostic loops. The CIs for estimates of loop closures when comparing telehealth visits with in-person visits for dermatology referrals and stress tests were wide, suggesting that our smaller sample sizes may have reduced our opportunity to identify important differences. Similarly, although the study used data from 2 different primary care settings, there were too few visits at the community site to detect significant differences. We lack data to fully explain why orders without a visit had completion rates that were similar to those of face-to-face visits. Whether the orders resulted from a patient telephone call or message was not available in the data set. In addition, the multivariable stepwise analysis suggests possible issues with residual confounding.

## Conclusions

In this cohort study, completion rates for diagnostic tests and referrals were low for all treatment modalities at 2 primary care sites, but worse for telehealth. These results are among the first to document how telehealth affects patients’ likelihood of completing recommended tests and specialty referrals. Telehealth visits present novel opportunities to reduce diagnostic errors and may play an increasingly important role in clinical practice given the continued expansion of telehealth following the COVID-19 pandemic.

## Notes

Supplement 1.
Data Sharing Statement
