# Supplementary material for: Completion of Recommended Tests and Referrals in Telehealth vs In-Person Visits
Source: JAMA Netw Open. 2023 Nov 15;6(11):e2343417. doi: 10.1001/jamanetworkopen.2023.43417 (PMC10652149; doi:10.1001/jamanetworkopen.2023.43417)
Supplement: Supplement 1. — Data Sharing Statement [file jamanetwopen-e2343417-s001.pdf]

Data Sharing Statement

Zhong. Completion of Recommended Tests and Referrals in Telehealth vs In-Person Visits. *JAMA Netw Open*. Published November 16, 2023. doi:10.1001/jamanetworkopen.2023.43417

Data
Data available: No
